# Health Care Utilization and Perceived Quality of Care in a Colombian Indigenous Health Organization

**DOI:** 10.1177/01632787241288225

**Published:** 2024-10-04

**Authors:** Aynslie Hinds, Beda Suárez Aguilar, Yercine Duarte, Dorian Ospina, John Harold Gómez Vargas, Javier Mignone

**Affiliations:** 1 8665 University of Winnipeg, Canada; 2 Anas Wayuu, Colombia; 3 8664 University of Manitoba, Canada

**Keywords:** indigenous health, indigenous healthcare governance, health care utilization, quality of care, Wayuu, Colombia, intercultural health

## Abstract

Indigenous governance of health care has increasingly been advocated among Indigenous peoples in many countries. However, there is limited research that has empirically examined its benefits. In 2020/21, we conducted a survey of 2113 Indigenous Wayuu individuals in Colombia who received services from the Indigenous Wayuu led health care insurance organization Anas Wayuu and its network of service providers, and Wayuu individuals who received services from non-Indigenous health insurance organizations. We compared their health care utilization and perception of quality of care. A main finding of the study was that Anas Wayuu enrollees were more than twice as likely to access health care than enrollees from non-Indigenous health insurance organizations, even when controlling for the demographic and health characteristics. The study provided compelling evidence suggesting that Anas Wayuu, being an Indigenous led health organization improves access to, and quality of care, among Indigenous health service recipients.

## Introduction

Indigenous governance of health care has increasingly been advocated among Indigenous peoples in many countries (Lavoie et al., 2016). Some potential benefits of health care being planned, administered, and delivered through Indigenous led organizations are increased access to care, more culturally relevant care, and better satisfaction with care (Montenegro & Stephens, 2006). In essence, Indigenous governance of health care can arguably be considered a key factor in decreasing health inequities as it relates to Indigenous peoples. However, there is limited research that has empirically examined its benefits. A recent paper (Hinds et al., 2024) assessed the consistency between administrative health records and self-reported health status and health care use among Indigenous Wayuu in La Guajira, Colombia. However, it did not examine health care utilization and perceived satisfaction comparing Anas Wayuu enrollees and those enrolled in health insurance organizations not Indigenous led (i.e., non-enrollees).

The Andersen Behavioral Model of Health Services Use (ABM) (Andersen, 1968, 1995, 2008) postulates that “health services use can be predicted or explained by population characteristics, including individuals’ predispositions to use services, resources that enable or impede use, and their need for care. Predisposing characteristics pertain to sociocultural factors that preclude a person’s illness and include demographics (age and gender), social structure (education, ethnicity, culture, social interactions, and occupation) and health beliefs (attitudes, values and knowledge of health and health services)” (Isaak et al., 2020). One aspect of the model, the notion that formal and informal resources that facilitate or hinder the use of health services, operates under the assumption that “people must have the means and know-how to get to those services and make use of them” (Andersen, 1995). The ABM includes among the enabling factors financing and organizational factors. Individual financing factors refer “to the income and wealth at an individual’s disposal to pay for health services and the effective price of health care which is determined by the individual’s health insurance status and cost-sharing requirements” (Babitsch et al., 2012). For Anas Wayuu enrollees, the subsidized regimen under which they receive health coverage in large part counteracts this factor.

There are numerous studies of culturally relevant health initiatives in Latin America, albeit few that properly address empirical outcomes, in large part due to the lack of data needed for these types of studies (Patiño-Londoño et al., 2016). The purpose of our study, as part of a larger study on intercultural health (Hinds et al., 2024), was to examine the differences between Anas Wayuu enrollees and those enrolled in non-Indigenous led health care organizations in terms of utilization and satisfaction, empirically characterizing the health care utilization and perception of quality of care among Wayuu individuals. The research question we sought to answer was: What are the differences in health care utilization and perceived quality of care among Wayuu individuals enrolled in Anas Wayuu and those enrolled in health organizations that are not Indigenous led?

## Method

The background information and the survey dataset are the same as in our previously published paper (Hinds et al., 2024), thus only some contextual and methodological information is summarized here. For details not reported here, please refer to the previous paper (Hinds et al., 2024).

### Context

The Wayuu People in Colombia live in the northeastern department (akin to provinces or states) of La Guajira with an estimated population of 380,500 (Departamento Administrativo Nacional de Estadística, 2019). Anas Wayuu is a Wayuu led health insurance organization created in 2001. Anas Wayuu is mostly managed by professionals of Wayuu origin who in large part are bilingual (Wayuunaiki and Spanish). As of 2020, Anas Wayuu provided health care coverage to approximately 220,000 people, 71% of whom are Indigenous Wayuu. Anas Wayuu contracts a network of health care providers. Through this network, Anas Wayuu provides health coverage in health promotion and disease prevention services, and primary, secondary, and tertiary health care (Mignone & Gómez Vargas, 2015). Indigenous led health care organizations, such as Anas Wayuu, can be considered inherently intercultural. Among their potential benefits are improvements in access to, and satisfaction with, care among Indigenous peoples.

### Dataset

Working together with Anas Wayuu we developed a 34-item questionnaire (researchers, Anas Wayuu staff, Anas Wayuu Knowledge Keeper). If respondents used health care, they were then asked about the quality of care. We defined quality of care as easiness in navigating the health care system and satisfaction with the encounters. Quality of care with respect to three aspects, administrative processes, medical consultations, and access to health services, was measured by enrollees that indicated not having experienced difficulties in navigating the system and by enrollees that attended clinical encounters and were satisfied with the encounter. Anas Wayuu’s cultural expert (a Wayuu Knowledge Keeper) played a key role in the development of the questionnaire. We hired a survey coordinator and four bilingual (Wayuunaiki/Spanish) Wayuu surveyors. Data collection started in full mid-September 2020. All surveys were conducted by cell phone, utilizing an app specifically developed for the study. Of a sample frame of 34,961 Wayuu individuals (90% of which were current enrollees and 10% past enrollees) between 18 to 80 years of age. 2160 surveys were completed. Once duplicates and poorly completed surveys were removed, the final sample consisted of 2113 individuals (Hinds et al., 2024).

### Data Analysis

Using SPSS v 25.0, we analyzed the data descriptively and inferentially. First, we examined the characteristics of the sample to assess how representative it was in comparison to the Anas Wayuu population. The results are presented as frequencies and percentages. Next, we compared the characteristics of Anas Wayuu enrollees and non-enrollees. The results are presented as frequencies and percentages, and chi-square tests were performed to determine if the groups differed significantly (*p* < .05). We followed this up with logistic regression to determine if enrollment status could be explained by differences in the characteristics (i.e., gender, age, region, languages spoken, and self-rated health). In the logistic regression model, we included the variables that were significant in the bi-variate analyses. Lastly, we divided the sample into two groups based on whether they had used healthcare (or not) in the past three years (self-reported). For those who had used healthcare, we determined the number and percentage who had positive, neutral, and negative experiences. Next, we compared the characteristics of those who had used healthcare and those who did not. The results are presented as frequencies and percentages, and chi-square tests were performed to determine if the groups differed significantly on several characteristics (*p* < .05). We followed this up with logistic regression to determine if healthcare use could be explained by enrollment status (while controlling for potential confounders). That is, we wanted to know if enrollment with Anas Wayuu facilitated use of healthcare (accounting for differences between the groups). We controlled for gender, age, region, languages spoken, and self-rated health, because they were statistically significant in the bi-variate analyses. Finally, we stratified the sample by gender and ran a logistic regression on each sub-sample.

## Results

### Characteristics of the Survey Respondents

The mean age of the survey participants was 39.6 years (SD = 15.9; Median age = 36; IQR = 25) (Appendix 1). The 65+ age group was overrepresented (8.8%) compared to 5% in La Guajira. There was a substantial female/male imbalance, as 64.7% of respondents were female and 35.3% male, whereas in La Guajira, 51.3% of the population are women and 48.6% are men. The percentages of those surveyed from the three Guajira large regions, Alta Guajira (32.4%), Media Guajira (62.3%), Baja Guajira (3.1%) correspond well with the regions where most of the Wayuu population resides. Less than half of respondents lived in urban areas and slightly more than half in rural areas, which suggests an over-representation of urban respondents (as per Anas Wayuu administrative data, 82% of Wayuu enrollees are rural residents). More than three quarters (80.5%) understand Spanish and less (63.9%) understand Wayuunaiki. Three quarters (77.0%) rated their health as excellent/very good/good, and the remaining (23.0%) rated their health as satisfactory/poor. About one-third (30.6%) reported having an infectious disease, and 26.7% of women reported having been pregnant in the last three years. Overall, the sample seemed representative of the population of interest, albeit with some over-representation of women and urban dwellers.

### Characterization of Anas Wayuu Enrollees and Non-Enrollees

Our comparison of enrollees and non-enrollees showed that Anas Wayuu enrollees that participated in the survey were on average older than non-enrollees (M = 40.37, SD = 16.31 vs. M = 34.85, SD = 12.41), which can also be seen in higher percentages of 45+ year olds among enrollees (26.7%) compared to non-enrollees (19.7%) (Appendix 1). The gender imbalance was greater among enrollees (66.9% females vs. 51.4% females). A higher percentage of Anas Wayuu enrollees were from Alta Guajira (34.9% vs. 17.3%), from rural areas (57.4 vs. 51.2%) and from the subsidized system (90.5% vs. 77.9%). As well, Anas Wayuu enrollees had a higher percentage of non-Spanish speakers (23.7% vs. 12.5%). A higher percentage of Anas Wayuu enrollees reported satisfactory/poor self-rated health (24.0% vs. 16.3%) and a higher percentage of the Anas Wayuu enrollees reported health conditions. One of the greatest differences was in relation to infectious diseases where 33.1% of Anas Wayuu enrollees reported having an infectious disease compared to 15.0% of the non-enrollees.

The logistic regression results used to determine the characteristics associated with enrollment status are presented in Table 1. The Nagelkerke R2 (pseudo-R2) for the full model is 0.078, suggesting that the model explained approximately 7.8% of the variation in the outcome. The Hosmer and Lemeshow test of goodness of fit suggests that the full model is a good fit to the data (χ2(8) = 10.15, *p*-value = .255). Anas Wayuu enrollees were twice as likely to be female, almost six times as likely to be aged 65 and older, and almost half as likely to speak Spanish (aOR = 0.54). Region of residence and self-rated health were not statistically significant when controlling for the other variables.

**Table 1.** ORs and 95% CIs for Being Currently Enrolled With Anas Wayuu

Variable	Response	Unadjusted OR (95% CI)^b^	Adjusted OR (95% CI)^c^
Gender	Male	Ref	Ref
	Female	**1.91 (0.49, 2.46)**	**2.07 (1.60, 2.70)**
Age group	18 – 29 years	Ref	Ref
	30 – 44 years	1.05 (0.79, 1.40)	1.08 (0.80, 1.44)
	45 – 64 years	**1.71 (1.23, 2.39)**	**1.68 (1.19, 2.36)**
	65+ years	**6.13 (2.66, 14.13)**	**6.16 (2.65, 14.31)**
Region^a^	Urban	Ref	Ref
	Rural	**1.29 (1.00, 1.65)**	1.25 (0.97, 1.61)
Speaks Spanish	No	Ref	Ref
	Yes	**0.46 (0.32, 0.66)**	**0.54 (0.37, 0.80)**
Speaks Wayuunaiki	No	Ref	Ref
	Yes	**1.31 (1.02, 1.69)**	1.10 (0.84, 1.44)
Self-rated health	Good to excellent	Ref	Ref
	Satisfactory/Poor	**1.62 (1.17, 2.25)**	1.39 (0.99, 1.95)

*Note.* Bold values indicate statistical significance at the 0.05 level.

^a^Missing *N* = 74.

^b^*N* = 2113.

^c^*N* = 2039.

### Health Care Utilization and Perceived Quality of Care

Many of the survey respondents (84.3%) reported using healthcare in the previous three years. Overall, respondents’ experience with healthcare was positive, regardless of the aspect of health service (Table 2). Anas Wayuu enrollees were more likely to report positive experiences than non-enrollees for each aspect of healthcare (*p* < .001).

**Table 2.** Quality of Experience With Aspects of Health Services in the Previous 3 Years

Variable	Response	Total (*N* = 1781)		Current enrollees (*N* = 1558)		Past enrollees (*N* = 223)	
		*N*	%	*N*	%	*N*	%
Administrative processes of the EPS	Positive	1497	84.1	1333	85.6	164	73.5
	Satisfactory	243	13.6	196	12.6	47	21.1
	Negative	41	2.3	29	1.9	12	5.4
Medical consultation through the EPS	Positive	1545	86.7	1371	88.0	174	78.0
	Satisfactory	208	11.7	166	10.7	42	18.8
	Negative	28	1.6	21	1.3	7	3.1
Access to health services through EPS	Positive	1436	80.6	1280	82.2	156	70.0
	Satisfactory	297	16.7	241	15.5	56	25.1
	Negative	48	2.7	37	2.4	11	4.9

The characteristics of individuals who did and did not access health services in the previous three years are presented in Table 3. Enrollees (85.7%) were significantly more likely to access health services than non-enrollees (75.9%). There were also statistically significant differences in use of healthcare services by gender, age, region of residence, self-rated health, languages spoken, some health conditions, and pregnancy.

**Table 3.** Characteristics of the Individuals Who Did and Did Not Access Healthcare Services in the Previous 3 Years (*N* = 2113)

Variable	Response	Used healthcare services				χ^2^	*p*-value
		Yes (*N* = 1781)		No (*N* = 332)			
		*N*	%	*N*	%		
Anas Wayuu enrollee status	Current	1558	85.7	261	14.3	**18.36**	**< 0.001**
	Past	223	75.9	71	24.1		
Gender	Male	596	80.0	149	20.0	**14.97**	**< 0.001**
	Female	1185	86.6	183	13.4		
Age group	18 – 29 years	622	83.7	121	16.3	**9.39**	**0.025**
	30 – 44 years	522	81.6	118	18.4		
	45 – 64 years	471	86.6	73	13.4		
	65+ years	166	89.2	20	10.8		
Region	Alta Guajira	553	80.7	132	19.3	**13.21**	**0.004**
	Media Guajira	1136	86.3	180	13.7		
	Baja Guajira	57	86.4	9	13.6		
	Other	35	76.1	11	23.9		
Type of region^a^	Urban	746	86.2	122	13.8	**4.39**	**0.036**
	Rural	955	82.8	198	17.2		
Type of insurance	Contributive	193	81.1	45	18.9	2.07	0.150
	Subsidized	1588	84.7	287	15.3		
Spanish	Speaks	1440	87.6	204	12.4	**61.03**	**< 0.001**
	Does not speak	341	72.7	128	27.3		
Wayuunaiki	Speaks	1055	80.4	257	19.6	**39.27**	**< 0.001**
	Does not speak	726	90.6	75	9.4		
Self-rated health	Good to excellent	1352	83.0	276	17.0	**8.25**	**0.004**
	Satisfactory/Poor	429	88.5	56	11.5		
Cardiovascular diseases	Yes	275	89.3	33	10.7	**6.80**	**0.009**
	No	1506	83.4	299	16.6		
Asthma, chronic or long-lasting bronchitis	Yes	64	84.2	12	15.8	0.00	0.985
	No	1717	84.3	320	15.7		
Gastritis	Yes	283	84.7	51	15.3	0.06	0.809
	No	1498	84.2	281	15.8		
Cancer	Yes	14	93.3	1	6.7	E <5	
	No	1767	84.2	331	15.8		
Diabetes	Yes	143	90.5	15	9.5	**4.99**	**0.026**
	No	1638	83.8	317	16.2		
Infectious diseases	Yes	560	86.7	86	13.3	**4.05**	**0.044**
	No	1221	83.2	246	16.8		
Pregnant	Yes	339	92.6	27	7.4	**23.22**	**< 0.001**
	No	1442	82.5	305	17.5		

*Note*. E < 5 means the expected value is less than 5. Bold values indicate statistical significance at the 0.05 level.

^a^*N* = 74 Missing type of region.

Logistic regression was performed to determine if enrollment status could explain use of healthcare in the three years prior to the survey. The Nagelkerke R2 (pseudo-R2) for the full model was 0.108, suggesting that the model explained approximately 10.8% of the variation in the outcome (using healthcare). The Hosmer and Lemeshow test of goodness of fit suggested the full model is a good fit to the data (χ2(8) = 7.02, *p*-value = .535). Anas Wayuu enrollees were 1.97 times as likely to have accessed healthcare in the last three years relative to individuals not currently enrolled with Anas Wayuu after controlling for demographic and health characteristics (Table 4). Females were 1.70 as likely than males to have accessed healthcare in the last three years. Older adults (65+) were more likely to access healthcare. Spanish speakers were more than twice as likely (aOR = 2.67) than non-Spanish speakers to access healthcare (i.e., Wayuunaiki only speakers were less likely to access healthcare). The adjusted Odds Ratio suggested that respondents with poor to satisfactory self-rated health were 1.42 times as likely to access health services compared to those with good to excellent health.

**Table 4.** ORs and 95% CIs for Accessing Healthcare in Previous 3 Years

Variable	Response	Unadjusted OR (95% CI)^b^	Overall^c^	Male^d^	Female^e^
			Adjusted OR (95% CI)	Adjusted OR (95% CI)	Adjusted OR (95% CI)
Anas Wayuu enrollee status	Past	Ref	Ref	Ref	Ref
	Current	**1.90 (1.41, 2.56)**	**1.97 (1.43, 2.71)**	**2.55 (1.63, 4.00)**	1.54 (0.95, 2.49)
Gender	Male	Ref	Ref	**-**	**-**
	Female	**1.62 (1.28, 2.05)**	**1.70 (1.32, 2.19)**	**-**	**-**
Age group	18 – 29 years	Ref	Ref	Ref	Ref
	30 – 44 years	0.86 (0.65, 1.14)	0.89 (0.66, 1.19)	0.77 (0.48, 1.24)	0.97 (0.67, 1.42)
	45 – 64 years	1.26 (0.92, 1.72)	1.38 (0.98, 1.94)	1.23 (0.73, 2.07)	1.49 (0.94, 2.35)
	65+ years	1.62 (0.98, 2.67)	**1.73 (1.02, 2.95)**	1.65 (0.76, 3.59)	1.80 (0.85, 3.81)
Region^a^	Urban	Ref	Ref	Ref	Ref
	Rural	**0.77 (0.60, 0.98)**	0.81 (0.62, 1.04)	0.69 (0.46, 1.02)	0.91 (0.65, 1.27)
Speaks Spanish	No	Ref	Ref	Ref	Ref
	Yes	**2.65 (2.06, 3.40)**	**2.67 (2.00, 3.58)**	**3.27 (2.06, 5.19)**	**2.30 (1.57, 3.36)**
Speaks Wayuunaiki	No	Ref	Ref	Ref	Ref
	Yes	**0.42 (0.32, 0.56)**	**0.61 (0.44, 0.83)**	**0.67 (0.42, 1.07)**	**0.56 (0.37, 0.86)**
Self-rated health	Good to excellent	Ref	Ref	Ref	Ref
	Satisfactory/Poor	**1.56 (1.15, 2.13)**	**1.42 (1.03, 1.97)**	1.29 (0.78, 2.14)	1.51 (0.98, 2.33)

*Note.* Bold values indicate statistical significance at the 0.05 level.

^a^Missing *N* = 74.

^b^*N* = 2113.

^c^*N* = 2039.

^d^*N* = 720.

^e^*N* = 1368.

The sample was stratified by gender, and we performed logistic regression for each sub-sample. The Nagelkerke R2 (pseudo-R2) for the male and female models were 0.138 and 0.072, respectively. The Hosmer and Lemeshow test of goodness of fit suggested the models for the sub-samples were a good fit to the data (Male: χ2(8) = 12.48, *p*-value = .131, Female: χ2(8) = 4.88, *p*-value = .771). Enrollment with Anas Wayuu was significantly associated with use of healthcare for males, but not for females. Males who were enrolled with Anas Wayuu were 2.55 times as likely to use healthcare than males who were not enrolled with Anas Wayuu, while enrollment status did not matter for females. Languages spoken were significantly associated with use of healthcare for both males and females. No other factors were significantly associated with healthcare use for either gender.

## Discussion

There were significant differences between Anas Wayuu enrollees and non-enrollees in their demographic characteristics (i.e., age, gender, region of residence, type of insurance, languages spoken) and health profiles. In general, Anas Wayuu enrollees were older, were less likely to speak Spanish, were more likely to report satisfactory or poor health, and were more likely to have a health condition. This was particularly striking for infectious diseases. The significance of these results is that Anas Wayuu tends to enroll and/or retain enrollees that health-wise are more vulnerable and potentially have higher healthcare needs.

Overall, there was a relatively high use of health care (84.3%) in the three years prior to the survey, and most people had a positive experience (medical consultations = 86.7%, administrative processes = 84.1%, accessing health services = 80.6%). Particularly noticeable was the very low reporting of negative experiences (2.3%, 1.6% and 2.7%, respectively). These findings compare favorably to surveys worldwide. For instance, a study conducted among the general population in Central and Eastern European countries found dissatisfaction ranging between 5% and 15% (Stepurko et al., 2016). As Jang and colleagues (2005) demonstrated, higher satisfaction is a facilitator of healthcare utilization.

An important finding when comparing Anas Wayuu male enrollees with male non-enrollees, was that those enrolled in Anas Wayuu were more likely (2x) to access healthcare. This effect was seen even when controlling for the demographic and health characteristics. This suggests that Anas Wayuu improves access to healthcare. The literature mentions racism and perceived discrimination (Allen et al., 2017; National Academies of Sciences, Engineering, and Medicine, 2018) as possible reasons for low rates of health care utilization. The fact that the biomedical health care system of Anas Wayuu is under the governance of Indigenous authorities, and a majority of Anas Wayuu’s staff and management are Indigenous, may explain how racism and perceived discrimination is likely not a barrier to utilization among Anas Wayuu enrollees. As well, since Anas Wayuu has strong engagement with the Wayuu communities in the region, including more presence of community health care workers, this is likely a factor in reducing sociocultural barriers to access

As expected, a higher percentage of women compared to men reported using healthcare services, as well as older age groups. Similar patterns of healthcare use are seen in other countries and regions (Bertakis et al., 2000). The percentages of urban and rural individuals who accessed health services did not differ substantially, which speaks well about access, given that barriers to access among rural populations are usually higher in many countries and regions. Not surprisingly, individuals with poorer self-rated health (i.e., those more likely to need it) were more likely to have used healthcare. Spanish speakers were more than twice as likely than non-Spanish speakers to access healthcare, which suggests room for improvement.

Within the ABM model, “organizational factors entail whether an individual has a regular source of care and the nature of that source. They also include means of transportation, travel time to and waiting time for health care” (Babitsch et al., 2012). Anas Wayuu has programs (e.g., intercultural hostels in urban centres, bilingual guides) that seek to minimize these potential barriers. At the contextual level, the ABM model considers the resources available within the community for health services, and “organization at this level refers to the amount, varieties, locations, structures and distribution of health services facilities and personnel” (Babitsch et al., 2012). The above-mentioned large network of health services that Anas Wayuu enrollees have access to relates to this factor. As well as the fact that Anas Wayuu is Indigenous led and the majority of staff and management are Wayuu. This may also explain a final important finding, that Anas Wayuu enrollees were more likely to report positive experiences, meaning higher satisfaction, than non-enrollees for each aspect of healthcare. These results fit with the ABM model’s prediction in relation to patient satisfaction (Swanson et al., 2003).

There are limitations to the study. One of the limitations was the use of cell phones for the survey. Although their use was quite successful, it introduced a bias of overrepresentation of urban dwellers due to less reliable connectivity in rural areas. The study sample was relatively well representative of the general Wayuu population in terms of age, the three La Guajira regions, and Wayuunaiki and Spanish speakers. However, there was a substantial female/male imbalance, which is not uncommon in community surveys. Despite the very low refusal rate (17.2%), suggesting a high willingness to participate in the survey among those who answered the phone, the large number of people that were not reachable may have been a factor in having a lower proportion of rural respondents than expected. Based on our knowledge of the La Guajira rural context, we are aware that Wayuu individuals living in non-urban areas tend to turn off their phones to save battery. Furthermore, connectivity in certain areas is less reliable.

## Conclusion

A main finding of the study was that Anas Wayuu enrollees were more than twice as likely to access health care than enrollees from non-Indigenous health insurance organizations, even when controlling for the demographic and health characteristics. Furthermore, that they were more satisfied with the services. This provides evidence suggesting that Anas Wayuu, being an Indigenous led health organization seems to improve access to, satisfaction, and quality of care among Indigenous health service recipients, supporting claims for Indigenous governance over healthcare.

## Appendix

### Appendix 1. Characteristics of Anas Wayuu Enrollees and Non-Enrollees

**Table:**

Variable	Response	All survey participants (*N* = 2113)		Anas Wayuu enrollee status				χ^2^	*p*-value
				Enrollee (*N* = 1819)		Non-enrollee (*N* = 294)			
		*N*	%	*N*	%	*N*	%		
Gender	Male	745	35.3	602	33.1	143	48.6	**26.79**	**< 0.001**
	Female	1368	64.7	1217	66.9	151	51.4		
Age group	18 – 29 years	743	35.2	617	33.9	126	42.9	**31.21**	**< 0.001**
	30 – 44 years	640	30.3	536	29.5	104	35.4		
	45 – 64 years	544	25.7	486	26.7	58	19.7		
	65+ years	186	8.8	180	9.9	6	2.0		
Region	Alta Guajira	685	32.4	634	34.9	561	17.3	**326.52**	**< 0.001**
	Media Guajira	1316	62.3	1140	62.7	176	59.9		
	Baja Guajira	66	3.1	45	2.5	21	7.1		
	Other	46	2.2	0	0.0	46	15.6		
Type of region^a^	Urban	886	43.5	743	42.6	143	48.8	**3.99**	**0.046**
	Rural	1153	56.5	1003	57.4	150	51.2		
Type of insurance	Contributive	238	11.3	173	9.5	65	22.1	**40.19**	**< 0.001**
	Subsidized	1875	88.7	1646	90.5	229	77.9		
Spanish	Speaks	1644	77.8	1387	76.3	257	87.4	**18.27**	**< 0.001**
	Does not speak	469	22.2	432	23.7	37	12.6		
Wayuunaiki	Speaks	1312	62.1	1146	63.0	166	56.5	**4.60**	**0.032**
	Does not speak	801	37.9	673	37.0	128	43.5		
Self-rated health	Good to excellent	1628	77.0	1382	76.0	246	83.7	**8.48**	**0.004**
	Satisfactory/Poor	485	23.0	437	24.0	48	16.3		
Cardiovascular diseases	Yes	308	14.6	286	15.7	22	7.5	**13.80**	**< 0.001**
	No	1805	85.4	1533	84.3	272	92.5		
Asthma, chronic or long-lasting bronchitis	Yes	76	3.6	65	3.6	11	3.7	0.02	0.886
	No	2037	96.4	1754	96.4	283	96.3		
Gastritis	Yes	334	15.8	299	16.4	35	11.9	**3.91**	**0.048**
	No	1779	84.2	1520	83.6	259	88.1		
Cancer	Yes	15	0.7	15	0.8	0	0.00	E <5	
	No	2098	99.3	1804	99.2	294	100.0		
Diabetes	Yes	158	7.5	154	8.5	4	1.4	**18.47**	**< 0.001**
	No	1955	92.5	1665	91.5	290	98.6		
Infectious diseases	Yes	646	30.6	602	33.1	44	15.0	**39.19**	**< 0.001**
	No	1467	69.4	1217	66.9	250	85.0		
Pregnant (% of females, excluding the 65+ age group) (*N* = 1262)	Yes	361	28.6	320	28.8	41	27.2	0.18	0.674
	No	901	71.4	791	71.2	110	72.8		

*Note.* ^a^N = 74 Missing Type of Region. Bold values indicate statistical significance at the 0.05 level.
